# Prevalence of Computer Vision Syndrome and Its Relationship with Ergonomic and Individual Factors in Presbyopic VDT Workers Using Progressive Addition Lenses

**DOI:** 10.3390/ijerph17031003

**Published:** 2020-02-05

**Authors:** Mar Sánchez-Brau, Begoña Domenech-Amigot, Francisco Brocal-Fernández, Jose Antonio Quesada-Rico, Mar Seguí-Crespo

**Affiliations:** 1 Doctoral Programme in Health Sciences, University of Alicante, 03690 San Vicente del Raspeig, Alicante, Spain; mdmsb7@gmail.com; 2 Department of Optics, Pharmacology and Anatomy, University of Alicante, 03690 San Vicente del Raspeig, Alicante, Spain; mm.segui@ua.es; 3 University Institute of Physics Applied to Sciences and Technologies, University of Alicante, 03690 San Vicente del Raspeig, Alicante, Spain; francisco.brocal@ua.es; 4 Department of Physics, Systems Engineering and Signal Theory, University of Alicante, 03690 San Vicente del Raspeig, Alicante, Spain; 5 Department of Clinical Medicine, University Miguel Hernández, 03550 San Juan de Alicante, Alicante, Spain; jquesada@umh.es; 6 Public Health Research Group, University of Alicante, 03690 San Vicente del Raspeig, Alicante, Spain

**Keywords:** computer vision syndrome, video display terminals, progressive addition lenses, presbyopia, workplace, ergonomics

## Abstract

This cross-sectional study estimated computer vision syndrome (CVS) prevalence and analysed its relationship with video display terminal (VDT) exposure, as well as sociodemographic, refractive, environmental, and ergonomic characteristics in 109 presbyopic VDT workers wearing progressive addition lenses (PALs). Usual spectacles were measured with a lens analyser, and subjective refraction was performed by an optometrist. CVS was measured with the CVS-Q©. VDT exposure was collected. Ergonomic evaluations were conducted in a normal working posture looking at the screen. Air temperature and relative humidity were measured (thermohygrometer), and illumination was measured (luxmeter). Descriptive analysis and differences in CVS prevalence, as a function of the explanatory variables, were performed (chi-square test). Multivariate logistic regression was used to identify factors associated with CVS (OR and 95% CI). The mean age was 54.0 ± 4.8 years, and 43.1% were women. The mean hours of VDT use at work was 6.5 ± 1.3 hours/day. The prevalence of CVS was 74.3%. CVS was significantly associated with women (OR 3.40; 95% CI, 1.12–10.33), non-neutral neck posture (OR 3.27; 95% CI, 1.03–10.41) and altered workplace lighting (OR 3.64; 95% CI, 1.22–10.81). Providing training and information to workers regarding the importance of adequate lighting and ergonomic postures during VDT use is advised to decrease CVS and increase workplace quality of life.

## 1. Introduction

The development of new information and communication technologies (ICTs) has led to an increase in the use of video display terminals (VDTs) in work environments. According to the Sixth European Working Conditions Survey [1], more than half of European workers use VDTs at work, and 37% use VDTs at least three-quarters of the workday. Finance, public administration and education are the economic sectors that use VDTs the most. Thus, among the challenges included in the EU Strategic Framework on Health and Safety at Work 2014–2020 [2] is the study of emerging risks, particularly those arising from new technologies.

In many studies, VDT exposure at work has been associated with an increase in visual complaints. Ocular and vision-related symptoms that result from the prolonged use of desktop and laptop computers, tablets, e-readers, and smart phones are known as computer vision syndrome (CVS) [3,4]. CVS depends on numerous factors derived from the demands of tasks, the characteristics of the workplace and the visual characteristics of the worker [5]. The eyes must focus on the screen, the documents, and the keyboard with continuous changes in accommodation and convergence [6]. In addition, good coordination of eye movements is required for merging images from both eyes, as is adequate binocular vision [7]. However, the concentration usually involved in reading tasks causes a reduced frequency and amplitude in blinking, which increases the tear film evaporation altering the ocular surface and leading to discomfort [8]. Additionally, poor lighting, screen reflections, inappropriate working distances and postures, and certain temperature and humidity conditions can increase this symptomatology. Sex, age, some pharmacological treatments, uncorrected refractive errors, and even the type of optical compensation may also be influential [3,4].

The studies published so far have the main limitation of evaluating CVS through unvalidated unstructured questionnaires, which include different symptoms according to the author and imprecise definitions of when a worker should be considered symptomatic [9,10,11], enormously compromising the findings. In Spain, a questionnaire with adequate psychometric properties was designed and validated in 2015 to measure CVS in workers exposed to VDTs [12]. Having this instrument facilitates more rigorous new research, particularly in those groups that are especially vulnerable [13].

Workers with presbyopia have special visual limitations in the use of VDTs due to their reduced accommodative capacity [14]. Progressive addition lenses (PALs) provide presbyopes with good vision at any distance, which makes them suitable for working with digital devices; however, their optical design can condition posture, angles and distance with respect to the screen [15]. It would be interesting to study the ocular and visual self-reported symptomatology by VDT workers that use this type of lenses and determine the associated factors.

Taking these considerations into account, the aim of our study was to estimate the prevalence of CVS in a sample of presbyopic VDT workers who wear PALs and to analyse its relationship with VDT exposure and sociodemographic, refractive, environmental and ergonomic characteristics.

## 2. Materials and Methods

### 2.1. Design and Setting

A cross-sectional study was conducted with a sample of presbyopic VDT workers at the University of Alicante (UA), Spain. The information was collected between January and May 2018. Neither refractive nor environmental and ergonomic conditions were modified in the study. Thus, the current state of the workers in the workplace was analysed.

### 2.2. Sample Selection

From a population of 1934 UA workers over 45 years of age, a representative sample of 114 individuals was calculated through GRANMO version 7.12 [16] to estimate a 50% predictive prevalence [13], with a 95% confidence interval and an accuracy of ±10 percentage points, considering an exclusion percentage of approximately 20%.

The inclusion criteria were workers who used a computer more than 4 hours/day during their workday and at least 5 days/week [5], habitual users of PALs in the workplace, and individuals with corrected binocular visual acuity (VA), at far and near distance, to at least 0.0 logMAR. Excluded were those who wore contact lenses, who had ocular surgery and who had ocular pathology or eye/systemic treatment in the 3 months prior to the study. Also excluded were those who regularly used a laptop (the inclination of the screen could vary at every moment).

E-mail was used to provide information about the study. A random selection was made from people interested in participating. Simple random sampling was performed.

### 2.3. Ethics

The study was approved by the Ethics Committee of UA (UA-2017-09-13), and the principles of the Declaration of Helsinki and the regulations on personal data protection were followed. All participants signed an informed consent form.

### 2.4. Data Collection

#### 2.4.1. Variables Related to Eye Health

An initial anamnesis was performed with questions about age, sex, ocular and systemic history/treatment. The usual spectacles were measured with the VISIONIX VX40 lens analyser (Luneau Technology, Chartres, France). Non-cycloplegic refraction was performed using retinoscopy followed by subjective refraction, and monocular and binocular visual acuity (VA) at far and near distances was measured. All these measures were carried out by a certified optometrist, who determined if the worker could be included in the study. The refraction clinical notation (S: sphere, C: cylinder, α: axis) was transformed into power vectors (M: spherical equivalent, J0 and J45: Jackson cross-cylinders), and the defocus (overall blurring strength) was calculated using the following formula: B = (M^2^ + J0^2^ + J45^2^)^1/2^ [17]. Each participant’s spherical equivalent and cylinder were considered as the mean M and as the mean C of the 2 eyes, respectively. Refractive error was categorized using the following definitions: myopia, mean M ≤ −0.75 D; hyperopia, mean M ≥ 1 D; and astigmatism, mean C ≥ 1 D [18]. Workers with ≤ 2 D addition were considered moderate presbyopes, and those with > 2 D were considered advanced.

CVS was measured using the Computer Vision Syndrome Questionnaire (CVS-Q©). This is a self-administered questionnaire, designed and validated by Seguí et al. [12], that evaluates the frequency and the intensity with which 16 symptoms are perceived over time with VDT use. From these data, a severity score is calculated for each symptom, and a final score is obtained; workers with a score ≥ 6 suffer CVS. The CVS-Q© has good psychometric properties derived from the Rasch Analysis, with sensitivity and specificity values greater than 70%. Also, the questionnaire has a good test-retest repeatability, evaluated for both the score using the intraclass correlation coefficient (ICC = 0.802; 95% CI, 0.673–0.884) and the diagnosis of CVS using Cohen’s kappa (ĸ = 0.612; 95% CI, 0.384–0.839). The area under the ROC curve is 0.826 (*p* < 0.001), indicating that the questionnaire has good diagnostic efficacy for detecting CVS. For all of these, it is a valid and reliable tool to evaluate the ocular and visual self-reported symptomatology by VDT workers.

#### 2.4.2. VDT Exposure and Environmental and Ergonomic Factors

Job category (TRS: Teaching and Research Staff or AS: Administrative Staff) and information regarding VDT exposure were collected: use in the workplace (hours/day, years, maximum continuous time, scheduled breaks, screen technology, and task type) and use for leisure.

**Figure 1:**
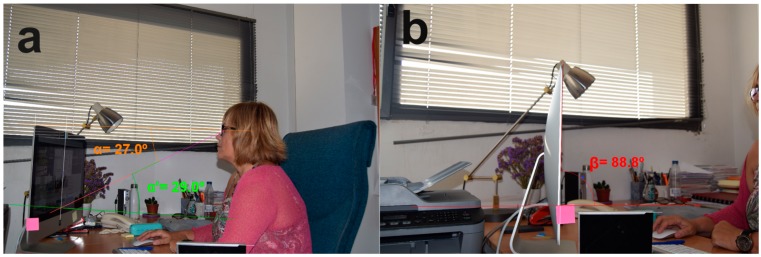
Drawing angles with QCad Trial 3.19.2 to calculate eye to screen angle α, vision angle α′ (**a**) and screen tilt angle β (**b**).

For the ergonomic evaluation, the workers were instructed to sit in a normal working posture and look at the screen. The viewing distance between the worker’s eyes and the centre of the screen was measured, and neck posture (neutral or altered) was analysed by direct observation of the worker according to standards [19]. Two photographs were taken with a Nikon D3300 camera, one from the sagittal plane of the seated worker and another from the plane perpendicular to the VDT (Figure 1a,b, respectively). Subsequently, the following were calculated using the software Qcad Trial 3.19.2 [20]: the eye to screen angle (α) formed between the horizontal from the eye and the line joining the eye with the lower centre of the screen; the vision angle (α′) formed by the last line described and the line perpendicular to the screen; and the screen tilt angle with respect to the horizontal (β).

Air temperature and relative humidity were measured with a thermohygrometer (PCE-WB 20SD). Illumination was measured using a luxmeter (ISO-TECH ILM 1337) in 3 positions: left, centre and right of the working area with a distance between extreme positions not exceeding 117 cm (maximum width of the working area [21]); the average of these 3 measurements was recorded. In all cases, the measurements were performed with the instruments supported on the table and with the worker sitting in their usual place for the time necessary for the values to stabilize. In addition, the following were recorded: if there was a direct glare over the eyes and reflections on the screen, if the workers used local (or task) lighting, and if the workers had the air conditioning turned on. Finally, the workers were asked to provide their subjective opinion on thermal comfort and lighting.

VDT exposure and environmental and ergonomic factors were collected through interviews and visits to the real workplace of the subjects by two researchers who received training in occupational ergonomics.

Normal parameters were considered a viewing distance between 45–75 cm, α < 60° and α′ < 40° [22]. The values considered normal for the ergonomic and environmental parameters were those reported by Spanish [23] and European [24] regulations. In cases where such regulations required technical criteria for interpretation, the values recommended by the technical guide [5] and the standard [19] were used as references. Thus, lighting between 500–1000 lux (considering high/very high visual requirements of the job) was considered normal [23] (the minimum recommended value is 500 lux [5]). Air temperatures between 20–24 °C for measurements taken from January to March and between 23–26 °C for measurements from April to May and relative humidity between 45–65% for any season of the year were considered normal [5]. The criteria to consider VDT adjustment adequate were the possibility of changing orientation and tilt at will and not having reflections that would bother the worker [24]. Finally, the neck posture should be neutral, what is considered normal or non-altered, avoiding flexion, extension, inclination and/or rotation thereof [19]. If these criteria were not met, the variables were considered altered.

### 2.5. Data Analysis

A descriptive analysis of all the variables studied was performed using absolute frequencies and proportions for the qualitative variables and averages (mean), standard deviations (SD) and ranges for the quantitative variables.

Paired samples Student’s *t*-test was applied to evaluate changes between usual spectacles and refraction dioptric power for the quantitative refractive variables and McNemar’s test for qualitative refractive variables. To detect differences in the prevalence of CVS as a function of individual characteristics and the workplace, a comparison of proportions was performed with the chi-square test. The magnitudes of the bivariate associations of the prevalence of CVS in the different categories of the explanatory variables were calculated using odds ratios (OR) and their confidence intervals at 95% (95% CI) estimated by binary logistic regression models. To identify the factors associated with CVS, a multivariate logistic model was used, estimating the OR of the association and its 95% CI. The stepwise selection of variables was performed based on AIC (Akaike information criterion). Goodness of fit indicators of the model are shown: chi^2^ value of the likelihood ratio test (LRT) and the associated *p*-value and the area under the ROC curve (AUC) and its 95% CI. A *p*-value < 0.05 was considered statistically significant. SPSS v.24 [25] and R v.3.5.1 [26] were used.

## 3. Results

A total of 109 workers participated in the study (Table 1). The mean age of the participants was 54.0 ± 4.8 years (range, 46 to 69), 43.1% were women, and 56.9% were AS. The mean use of VDT at work was 6.5 ± 1.3 hours/day (range: 4–10) and 23.3 ± 5.6 years (range: 8–35). A total of 76.1% of the participants used VDTs at work 5 days/week, while the rest used VDTs more days/week. A total of 53.2% of the participants had a work time at the VDT greater than 1 hour, and only 7.3% performed scheduled breaks during their use; the average break duration was 5.75 ± 2.96 minutes (range: 1–10). The mean total use of VDTs (work and leisure) was 8.7 ± 1.8 hours/day (range, 4.7 to 13). The display technologies most used were LED backlight (42.2%) and TFT (38.5%). The percentage of time that workers spent on average on different tasks was: 76.5% for text editing, 8.0% for graphics and figures, 2.6% for engineering projects, 4.3% for computer programming, and 8.5% for other tasks.

In general, the values obtained from the ergonomic and environmental parameters were normal according to Spanish [23] and European [24] regulations. However, the measured values corresponding to relative humidity (41.5 ± 5.6%) and illumination (489.6 ± 297.4 lux) were closer to the low end of the range of recommended values [5]. These changes were the most frequent in the workplaces (relative humidity 74.3%; illumination 70.6%), followed by adjustment of the display (44.0%) and neck posture (31.2%). No worker had an altered eye to screen angle or vision angle (Table 2). A total of 6.4% of the participants presented flexion, 20.2% extension and 7.3% neck rotation; sometimes the same worker had more than one altered neck posture. A total of 94.5% of the participants were not affected by a direct glare over the eyes.

The refractive characteristics of the VDT workers are shown in Table 3. For far vision, the mean B was between 2.50 and 2.75 D both in the OD and in the OS, without observing differences between the workers’ usual spectacles and the refraction performed by the optometrist. On the other hand, statistically significant differences were found in the addition (*p* < 0.001). For these reasons, the classification of refractive errors remained similar, in percentages, but more presbyopes were classified as advanced as a result of refraction (60.6% vs. 48.6%; *p* < 0.011). These differences in the addition were not related to a higher prevalence of CVS (*p* = 0.757).

**Figure 2:**
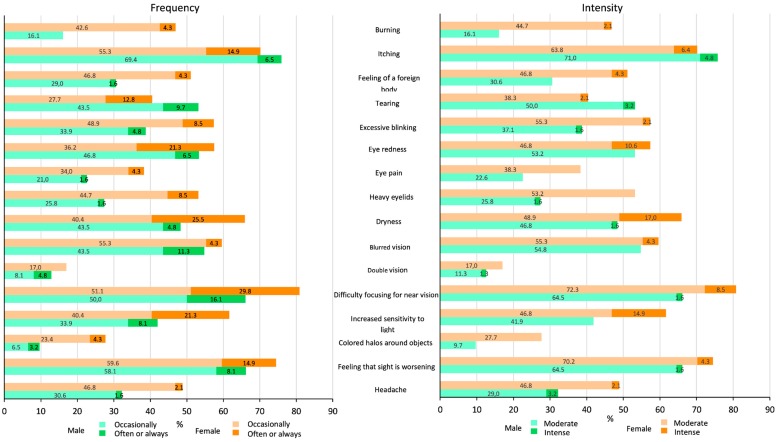
Percentage of workers (women and men) who present each symptom on the Computer Vision Syndrome Questionnaire (CVS-Q©) with a certain frequency and intensity.

The most frequent symptoms were itching (73.4%), difficulty focusing for near vision (72.5%) and feeling that sight is worsening (69.7%), followed by blurred vision, dryness, eye redness and increased sensitivity to light, with prevalences between 50%–57%. Less frequent symptoms were double vision and coloured halos around objects, with prevalences below 20%. Figure 2 shows that, in general, women present symptoms more frequently and intensely.

The prevalence of CVS was 74.3% (Table 4). No statistically significant differences were found between CVS and each of the explanatory variables, except for sex. Women had a higher prevalence than did men (85.1% vs. 66.1%; *p* = 0.025), presenting a higher association of suffering from CVS (OR = 2.93; 95% CI, 1.12–7.64; *p* = 0.028).

The results of the multivariate logistic analysis (Table 5) indicate that the factors that were associated with CVS in the proposed model were sex (OR = 3.40; 95% CI, 1.12–10.33; *p* = 0.031); non-neutral neck posture (OR = 3.27; 95% CI, 1.03–10.41; *p* = 0.045) and altered lighting (OR = 3.64; 95% CI, 1.22–10.81; *p* = 0.020). The variables age, myopia, total use of VDT and job category were adjustment variables. The model fit well with the data, with an LRT of 21.2 (*p* = 0.003) and an AUC of 0.78 (95% CI, 0.68–0.87).

## 4. Discussion

These results show a high prevalence of CVS (74.3%) in the sample of presbyopic VDT workers studied. For the environmental variables, a high percentage of workers (70%–75%) were found to be working with inadequate lighting and relative humidity. Only 56% of the sample had a screen that could be oriented, tilted and without reflections, as established by regulations. Neck posture in 31.2% of the sample was not neutral (flexion, extension and/or rotation of the neck). The optical compensation of workers with their usual spectacles (PALs) was adequate for far vision; however, 16.5% lacked addition in near vision. After the multivariate analysis, the three factors that were associated with an increase in CVS were being female, maintaining a non-neutral neck posture in front of the screen and having low lighting in the workplace.

It is difficult to compare these results with those from previous studies because many studies report the prevalence of one or more individual symptoms without providing information about CVS as a global construct. Even so, the high prevalence of CVS observed in our study confirms what the literature indicates: presbyopia is an important factor associated with asthenopia, given that the effort to focus among presbyopic VDT users increases the stress on the already meager accommodative reserve [27,28,29]. However, other studies did not observe differences between presbyopes and non-presbyopes, as in the study by Tomei et al. [30], in which an asthenopia prevalence of 46.2% was observed in those over 40 years, or in the study by Tauste et al. [13], in which a CVS prevalence of 51% was reported for patients over 45 years of age; in both cases, the prevalence was much lower than that estimated in our study. In the study by Tomei et al., asthenopia was measured with an unvalidated ad hoc questionnaire that evaluated eight symptoms (compared to 16 in the CVS-Q©), and the VDT workers were all men; the sample in our study included 43.1% women, which could partially explain the different prevalences, especially if one takes into account the association of sex with CVS. In contrast, in the study by Tauste et al. in which the CVS-Q© was also used, 52% were women, and possibly different environmental and ergonomic conditions in the workplace (factors not analysed by these authors) could explain the different results obtained between the studies.

Additionally, there are several studies that warn that working with VDTs in low light can cause the eyes to tire gradually [4,31,32]. Proper lighting is essential to prevent visual fatigue, which positively contributes to performance, safety, health and well-being at workplace as well as a reduction in work accidents and absenteeism [33]. The recommendations on lighting levels vary according to the tasks that are performed; thus, 300 lux is found most comfortable for entry of figures, whereas 500 lux is comfortable for edition of text because the latter is a task with greater visual demands [34]. A low relative humidity (below 40%) together with a high temperature increase the evaporation of tear film, producing hyperosmolarity and ocular dryness [27]. In our study, workers used VDTs for text editing on average 76.5% of the time; therefore, most lighting levels at workplaces were considered outside of the recommended range. However, this change is characterized by an important dispersion of the results (489.6 ± 297.4 lux) around 500 lux [5]. So, a more detailed analysis of the lighting in each workplace would be necessary to adopt measures and draw specific conclusions. In relation to relative humidity, most of the workplaces were considered inadequate, taking as a reference the recommended values to prevent dry eyes and mucous membranes [5]. However, similar to that indicated for lighting, the measured values corresponding to relative humidity (41.5 ± 5.6%) are around the lower end of the range (45%) considered as reference [5]; therefore, there is also uncertainty about limits considered as inadequate for specific jobs. Temperature was only higher than recommended in four cases. Overall, 83.5% of the workers subjectively rated lighting as comfortable, and 74.3% subjectively rated thermal comfort as adequate. Notably, all the facilities at UA that were analysed have air conditioning that can be adjusted, and 46.8% of the workers were using it at the time of the measurements. Moreover, only three workers habitually used local (or task) lighting to reinforce the ceiling lighting fixtures, and a direct glare was only observed in six cases. All of these factors support the adequate subjective assessment of worker comfort. Thus, in relation to the criteria of normality considered in this study for lighting and relative humidity in the workplace with VDTs, future studies should be developed with which to deepen the definition, analysis and evaluation of the most appropriate intervals for reducing CVS.

The most frequently neck posture alteration was extension (22 workers). However, the eye to screen angle and the vision angle were within the values that the standard recommends [19]. Most likely, an insufficient addition in PALs observed after refraction performed by the optometrist is the origin of a posture with the neck tilted backward (extension) in which the eyes of the worker seek the area with greater positive power of their usual spectacles to see the screen clearly. Moreover, the high percentage of altered VDT adjustment in this study (44%) could induce some of the inadequate neck postures observed. For the angles, in a study that compared the placement of a display in pre-presbyopes and presbyopes with multifocal lens correction, the latter selected a lower viewing height; as a result, the eye to screen angle for presbyopes was significantly lower than that for pre-presbyopes [35]. This suggests that ergonomic display placement recommendations should be different for computer users wearing multifocal correction for presbyopia [36]; therefore, it would be advisable to update the ergonomic reference criteria in this regard.

The correction of refractive errors and presbyopia is considered an important factor to ensure subjective visual comfort and decrease self-reported symptomatology by VDT workers [27,28,29]. Especially critical is uncorrected astigmatism, which influence vision even when it is on the order of 0.50–1.00 D [37,38]. In our study, uncorrected refractive errors were not observed in far vision but in near vision. Although there was no direct relationship between the errors in the addition and an increase in CVS, they could indirectly be influencing the adoption of bad neck postures, as mentioned above, which increases CVS according to our findings (OR = 3.27; 95% CI, 1.03–10.41; *p* = 0.045). In this sense, Daum et al. [39] estimated a highly favourable cost-benefit ratio to employers that provided adequate refractive correction to their employees, which increased productivity by at least 2.5%, given that the symptoms can increase the number of failures committed, in addition to making necessary breaks more frequent [40,41,42,43].

As a result of the multivariate model, our study did not associate age with an increase in CVS. However, during the normal aging process there is a progressive loss of retinal image quality due to the increase in ocular aberrations and the amount of scattered light produced by the ocular lens that invariably becomes less transparent with age. It should be considered that the visual quality of perceived images depends on the pupillary diameter, which is dependent on age. The results reported by Guillon et al. [44] note that both age and refractive status were found to affect pupil size, with larger pupils measured for younger patients and myopes. Additionally, Winn et al. [45] suggest that the size of the pupil decreases in an almost linear manner with increasing age; however, they did not find a relationship with refractive error. Senile miosis makes it difficult to see, especially in low ambient lighting because the pupils are less reactive. Therefore, it could be thought that, in our study, the visual symptomatology and consequently CVS should increase with age. However, a smaller pupil may reduce ocular aberrations and the amount of light scatter [46], while improving the depth of focus, which would reduce the accommodative strain. We think that these may be some of the reasons why the literature presents contradictory findings on the evaluation of the relationship between CVS and age [47].

The higher prevalence of CVS in females observed in our study (85.1% women vs. 66.1% men) is also consistent with numerous previous reports [13,48,49,50,51,52]. This association with sex could be related to dry eye [53,54,55,56]. Notably, there are several authors who have determined 2 broad categories of symptoms in the diagnosis of CVS [49,57]: a first group of internal symptoms (including blurred vision, diplopia or headache) generally caused by refractive, accommodative or vergence anomalies and a second group of external symptoms related to dry eye (including burning, itching, dryness or tearing). Female sex is one of the most consistent risk factors in the development of dry eye, becoming more significant with age [58,59]. Two studies conducted with North American populations of more than 39,000 women [55] and 25,000 men [56] showed that women had a 70% higher risk of dry eye than did men. There are also abundant studies that point to the biological plausibility of this association, such as those related to hormonal differences [60], differences in sensitivity [61], and differences in autoimmunity [62]. Overall, sex steroids (androgens, oestrogens) and thyroid hormones play major roles in the regulation of the ocular surface and adnexal tissues, and in the difference in dry eye prevalence between women and men [63], treatment with postmenopausal hormone therapy also increases the risk [64]. In our study, the population was older than 45 years; therefore, women were in the perimenopausal and menopausal age group, which can support our findings, keeping in mind that workers who followed systemic treatment in the three months prior to the study (including hormones) were excluded to avoid bias.

The job categories included in the multivariate model presented in our study may have influenced the results regarding CVS for several reasons. On the one hand, there is a higher percentage of women in administrative positions; 68.1% of women work as AS, compared to 48.4% of men. On the other hand, the different patterns of VDT use at work in these job categories could be conditioning the ocular and visual self-reported symptomatology. In a recent article by Soria-Oliver et al. [65], which classified 1259 workers from different Spanish productive sectors according to patterns of VDT use, based on the specific use of different devices (desktop or laptop computer, tablet, or smart phone) and their mobility dimension, the results showed that there are clear differences in the level of visual discomfort between the different patterns. In our case, although all VDT workers use a desktop computer in the university, due to the particularities of their work, the AS present less flexibility in terms of mobility in the workplace than do the TRS, given that the latter combine office work with teaching and laboratory research. Our results also include myopia as an adjustment variable. Close work can also induce transient myopia. Luberto et al. [66] even suggested that the use of a temporary myopic shift can be a reliable objective assessment tool for VDT-related visual fatigue. However, in a 10-year follow-up study published in 2019, no relationship between visual fatigue and a refractive state was found in VDT operators [31]. Therefore, further research should be conducted. Notably, in the sample studied by us, there were many more myopic men (61.3%) than women (36.2%).

However, it is surprising that the hours of VDT use at work were not associated with CVS in our study, in contrast to what is established in the literature [31,67,68,69], although there are also some exceptions [9,11]. One of the possible difficulties regarding this issue is that workers could have overestimated their time of VDT use, both at work and outside work. We think that, today, the almost continuous use of digital devices makes it difficult to quantify adequately and even separate the time of VDT use dedicated to work and that dedicated to other activities. Consequently, the effects on the visual health of workers (presence of CVS) due to prolonged VDT use should be assumed to be cumulative. In the future, it will be necessary to develop tools to quantify exposure to VDTs much more precisely in different areas of work and leisure and with different devices used. Only in this way will it be possible to reliably analyse the association of CVS with the time of VDT use.

Another limitation of our study was not being able to have more characteristics regarding the usual spectacles of the workers; it was only known that they were not occupational lenses and that their power was measured with a lens analyser. Today, PALs with different designs are marketed, with more or less broad fields of vision in intermediate and near vision [70], which could influence both ocular and visual self-reported symptomatology [70,71] and the neck posture adopted by workers, with more or less extension depending on the positive power increase along the corridor [32,72]. The possible effects of other factors that the literature relates to CVS, such as changes in binocular vision [3,7], the state of tear film or the ocular surface [8], or even job stress or exhaustion [9,73] have not been addressed. We can guarantee that we studied a sample of workers with good visual function (corrected binocular VA to at least 0.0 logMAR) and without severe dry eye problems nor ocular pathology or eye/systemic treatment in the three months prior to the study. The use of a validated questionnaire to evaluate CVS constitutes a great strength of this study and differentiates it from previous studies [9,31,65,71], providing reliability to the findings.

Finally, since this study shows a high prevalence of CVS in presbyopic PAL wearers who use VDT, it is advisable to include possible recommendations in order to reduce these symptoms. Both periodic visual exams that ensure update optical compensation and most convenient lens designs according to the demands are essential [4]. Particularly, the optical advantages of occupational lenses reduce CVS in relation to PALs in presbyopic VDT workers [70,71,74]. Correct physical ergonomics should be maintained in the workplace and avoid postural distortions that often lead to pain in shoulders, neck and upper back areas [75]. Likewise, the illumination in the workplace should be adequate to the tasks to be performed, with high visual demands in the case of these workers [3]. 

The specific preventive measures according to current Spanish and European regulations will be subject to future studies, which would provide the necessary knowledge for their update. In addition, lifestyle and biometric data could be collected in the future to establish a possible association with CVS [76]. 

## 5. Conclusions

Presbyopic PAL wearers are a group of workers especially susceptible to suffering ocular and visual symptoms related to the use of VDTs, with a prevalence of CVS greater than 70%. In this group, an association of CVS with the female sex is observed, with illumination values different from those recommended and neck postures different from neutrality.

In general, providing training and information to workers regarding the importance of maintaining adequate levels of lighting and ergonomic postures is advisable to reduce the discomfort caused by CVS during VDT use and increase the quality of life at the workplace, which can also have significant organizational benefits for businesses.

## Figures and Tables

**Table 1:** Sociodemographic and VDT exposure characteristics of the sample (*n* = 109).

Variables	*n*	%
Age (years)		
*46–50*	26	23.9
*51–52*	22	20.2
*53–55*	30	27.5
*56–69*	31	28.4
Sex		
*Male*	62	56.9
*Female*	47	43.1
Job category		
*TRS*	47	43.1
*AS*	62	56.9
Use of VDT at work (hours/day)		
*≤6*	53	48.6
*>6*	56	51.4
Use of VDT at work (years)		
*≤20*	37	33.9
*21–29*	50	45.9
*≥30*	22	20.2
Continuous work time at VDT (minutes)		
*≤60*	51	46.8
*61–120*	44	40.4
*>120*	14	12.8
Scheduled breaks during VDT work		
*No*	101	92.7
*Yes*	8	7.3
Total use of VDT (hours/day)		
*≤8*	49	45.0
*>8*	60	55.0
Screen technology		
*LED backlight*	46	42.2
*LED*	14	12.8
*LCD*	5	4.6
*TFT*	42	38.5
*LCD-TN*	2	1.8

Abbreviations: VDT: video display terminal; TRS: Teaching and Research Staff; AS: Administrative Staff; LED: Light Emitting Diode; LCD: Liquid Cristal Display; TFT: Thin Film Transistor; TN: Twisted Nematic. The categories of the variables are in Italic type.

**Table 2:** Environmental and ergonomic characteristics and percentage of VDT workers with altered variables (*n* = 109).

**Variables**	**Mean**	**SD**
Viewing distance (cm)	67.6	8.9
Eye to screen angle α (°)	26.8	5.7
Vision angle α′ (°)	21.1	5.2
Screen tilt angle β (°)	96.4	6.1
Air temperature (°C)	22.7	1.7
Relative humidity (%)	41.5	5.6
Illumination (lux)	489.6	297.4
**Altered variables**	***n***	**%**
Viewing distance	18	16.5
Eye to screen angle α	0	0.0
Vision angle α′	0	0.0
Air temperature	19	17.4
Relative humidity	81	74.3
Illumination	77	70.6
VDT adjustment	48	44.0
Neck posture	34	31.2

Abbreviation: VDT: video display terminal.

**Table 3:** Dioptric power of usual spectacles and refractive characteristics of the sample (*n* = 109).

Variables	Usual Spectacles *		Refraction **		*p*-Value ***
	Mean	SD	Mean	SD	
B (D)					
*OD*	2.67	1.82	2.71	1.78	0.161
*OS*	2.56	1.86	2.61	1.84	0.085
Addition (D)	2.06	0.44	2.20	0.30	**<0.001**
	***n***	**%**	***n***	**%**	***p*** -Value ****
Refractive error					
*Emmetropia*	23	21.1	23	21.1	1.000
*Myopia*	55	50.5	55	50.5	1.000
*Hyperopia*	31	28.4	31	28.4	1.000
*Astigmatism*	39	35.8	32	29.4	0.392
Presbyopia					
*Moderate*	56	51.4	43	39.4	**0.011**
*Advanced*	53	48.6	66	60.6	

Abbreviations: OD: right eye; OS: left eye; B: overall blurring strength. * Power measured with lens analyser. ** Non-cycloplegic refraction performed by the optometrist. *** Paired Student’s *t*-test. **** Mc Nemar’s test. Bold results are statistically significant. The categories of the variables are in Italic type.

**Table 4:** CVS prevalence and its relationship with VDT exposure, sociodemographic, refractive, environmental, and ergonomic characteristics.

Variables	*n*	P	*p*-Value *	OR	95% CI	*p*-Value **
TOTAL	81	74.3				
Age (years)						
*46–50*	19	73.1	0.987	1		
*51–52*	16	72.7		0.98	0.27–3.52	0.978
*53–55*	23	76.7		1.21	0.36–4.06	0.757
*56–69*	23	74.2		1.06	0.33–3.46	0.924
Sex						
*Male*	41	66.1	**0.025**	1		
*Female*	40	85.1		2.93	1.12–7.64	**0.028**
Job category						
*TRS*	31	66.0	0.082	1		
*AS*	50	80.6		2.15	0.90–5.15	0.085
Use of VDT at work (hours/day)						
*≤6*	36	67.9	0.138	1		
*>6*	45	80.4		1.93	0.81–4.64	0.141
Use of VDT at work (years)						
*≤20*	26	32.1	0.782	1		
*21–29*	38	46.9		1.34	0.51–3.49	0.550
*≥30*	17	21.0		1.44	0.42–4.88	0.560
Continuous work time at VDT (minutes)						
*≤60*	40	78.4	0.219	1		
*61–120*	29	65.9		0.53	0.21–1.33	0.175
*>120*	12	85.7		1.65	0.32–8.50	0.549
Scheduled breaks during VDT work						
*No*	74	73.3	0.677	1		
*Yes*	7	87.5		2.55	0.30–21.73	0.391
Total use of VDT (hours/day)						
≤8	39	79.6	0.254	1		
>8	42	70.0		0.60	0.25–1.45	0.257
Screen technology						
*LED backlight*	33	71.7	0.541	1		
*LED*	9	64.3		0.71	0.20–2.52	0.595
*LCD*	3	60.0		0.59	0.09–3.95	0.588
*TFT*	34	81.0		1.67	0.61–4.56	0.314
*LCD-TN*	2	100.0		-	-	-
Viewing distance (cm)						
*Non-altered*	69	75.8	0.417	1		
*Altered*	12	66.7		0.64	0.21–1.90	0.419
Eye to screen angle α (°)						
*Non-altered*	81	74.3	-	-	-	-
*Altered*	0	0.0				
Vision angle α′ (°)						
*Non-altered*	81	74.3	-	-	-	-
*Altered*	0	0.0				
Air temperature (°C)						
*Non-altered*	67	74.4	1.000	1		
*Altered*	14	73.7		0.96	0.31–2.96	0.945
Relative humidity (%)						
*Non-altered*	20	71.4	0.685	1		
*Altered*	61	75.3		1.22	0.47–3.20	0.686
Illumination						
*Non-altered*	20	62.5	0.069	1		
*Altered*	61	79.2		2.29	0.93–5.64	0.072
VDT adjustment						
*Non-altered*	44	72.1	0.557	1		
*Altered*	37	77.1		1.30	0.54–3.12	0.557
Neck posture						
*Non-altered*	52	69.3	0.077	1		
*Altered*	29	85.3		2.57	0.88–7.47	0.084
Ametropia						
*Emmetropia*	19	82.6	0.465	1		
*Myopia*	41	74.5		0.62	0.18–2.13	0.444
*Hyperopia*	21	67.7		0.44	0.12–1.65	0.224
Astigmatism						
*No*	54	70.1	0.121	1		
*Yes*	27	84.4		2.30	0.79–6.71	0.128
Presbyopia						
*Moderate*	31	72.1	0.669	1		
*Advanced*	50	75.8		1.21	0.51–2.89	0.669

Abbreviations: CVS: computer vision syndrome; P: prevalence; OR: odds ratio; CI: confidence interval; VDT: video display terminal; LED: Light Emitting Diode; LCD: Liquid Cristal Display; TFT: Thin Film Transistor; TN: Twisted Nematic. * Chi-square test. ** Univariate logistic regression analysis. Bold results are statistically significant. The categories of the variables are in Italic type.

**Table 5:** Factors associated with CVS: multivariate logistic regression model.

Variables	OR	95% CI	*p*-Value
Age	1.02	0.91–1.13	0.771
Sex			
*Male*	1		
*Female*	3.40	1.12–10.33	**0.031**
Myopia			
*No*	1		
*Yes*	1.57	0.57–4.23	0.386
Job category			
*TRS*	1		
*AS*	2.45	0.90–6.67	0.079
Total use of VDT (hours/day)			
*≤8*	1		
*>8*	2.59	0.96–6.98	0.061
Neck posture			
*Non-altered*	1		
*Altered*	3.27	1.03–10.41	**0.045**
Illumination			
*Non-altered*	1		
*Altered*	3.64	1.22–10.81	**0.020**

Abbreviations: CVS: computer vision syndrome; OR: odds ratio; CI: confidence interval; TRS: Teaching and Research Staff; AS: Administrative Staff. Bold results are statistically significant. The categories of the variables are in Italic type.
